# Evaluation of a long-term antimicrobial dental adhesive via *in vitro* biodegradation and *in vivo* rodent secondary caries models

**DOI:** 10.1016/j.dental.2025.08.017

**Published:** 2025-08-27

**Authors:** Cameron A. Stewart, Kimberly Ngai, Zach Gouveia, Sagar Rao, Dua Abuquteish, Andreas Mandelis, Yoav Finer

**Affiliations:** a Faculty of Dentistry, University of Toronto, Canada; b Institute of Biomedical Engineering, University of Toronto, Canada; c Department of Pediatric Laboratory Medicine & Pathobiology, Division of Pathology, The Hospital for Sick Children, Toronto, Canada; d Department of Microbiology, Pathology and Forensic Medicine, Faculty of Medicine, The Hashemite University, Zarqa, Jordan; e Center for Advanced Diffusion-Wave and Photoacoustic Technologies (CADIPT), Department of Mechanical & Industrial Engineering, University of Toronto, Toronto, Canada

**Keywords:** Antimicrobials, Dental materials, Dental caries, Biodegradation, Secondary caries

## Abstract

**Objectives:**

Bacterial-derived secondary caries is a primary cause of dental treatment failure at the artificial material-tissue interface. We previously developed ultra-long-term antimicrobial/antidegradative drug-silica particles (DSPs) to counter this interfacial failure. The aim of the current study was to evaluate a novel DSP-filled-adhesive system via *in vitro* and *in vivo* (rat) anti-secondary-caries studies.

**Methods:**

DSPs were incorporated into commercial total-etch dental adhesive at 10 % wt. to make DSP-SBMP. Interfacial specimens of DSP-SBMP-dentin and control SBMP-dentin were incubated 0- or 6-months in simulated salivary esterase, and subsequently with *S. mutans/L. rhamnosus* co-culture. Interfacial biomarkers were assessed via confocal microscopy and micro-computed-tomography. DSP-SBMP and SBMP were used to restore teeth in 16 SD rats in a 7-week split-mouth secondary caries study followed by clinical and μCT caries analysis and organ histology to assess biocompatibility.

**Results:**

*In vitro,* interfacial biofilm viability (-23.1 ± 4.3 %) and biomass (-19.2 ± 4.9) were reduced by DSP-SBMP, as was cavitated (-78.6 ± 13.8 %) and demineralized (-33.4 ± 8 %) volume (ANOVA, Tukey HSD, p < 0.05). *In vivo* clinically observed primary and secondary caries counts were reduced on DSP-SBMP-restored teeth (χ^2^ p < 0.05). No significant toxic effects were observed.

**Significance:**

This comprehensive *in vitro* and *in vivo* antimicrobial/antidegradative analysis of a new dental biomaterial, accurately modeling the chemical and biological environment these materials must perform in, provided comprehensive understanding of potential material performance that strongly supports continued development and clinical evaluation. The clinical relevance of the *in vitro* model used in this study was validated by the *in vivo* animal model and could be used to assess new dental biomaterials.

## 1. Introduction

Dental caries, which is the complex decay of teeth due to dietary, host and bacterial factors, is the most common chronic disease on the planet. Outcomes vary greatly between full restoration and pain/loss of function, depending on underlying patient factors and the treatments available [1]. Despite the frequency and severity of this disease, treatment methods for deep carious lesions have not significantly changed since the introduction of tooth-coloured resin restorations and their associated placement techniques more than 50 years ago [2]. Tooth colored methacrylate-based resins remain highly susceptible to bacterial, salivary, and host-immune biodegradation, and their bonded interfaces may be degraded and compromised by these processes through exposure and subsequent bacterial acidic attack, host-immune-, bacterial-, and to a lesser extent dentinal-collagenolytic activity [3,4]. These destructive processes lead to restoration failure due to detachment or secondary caries at the restoration-tooth interface, usually 5–7 years after placement [5]. In the United States, these failures are conservatively estimated to number 100–200 million and cost tens of billions to replace (however, it should be noted that recent US epidemiological data on recurrent caries is lacking [5,6]).

The development of new restorative materials capable of minimizing enzymatic degradation of restorative resins and eliminating bacteria from the restoration margins are two increasingly popular strategies towards reducing secondary caries formation [4,7]. Such emerging material developments, however, have been increasingly validated through short term preclinical aging using conditions and media with limited physiological relevance such as water or simple buffers [8]. These methodology shortcomings have been reinforced by recent attempts to standardize testing through ISO 3990:2023, which may help when comparing materials between simple studies but does not capture clinical efficacy. Additionally, studies involving bacteria are typically only utilized to examine the immediate effects on microbial proliferation. In some cases, such as with surface-bound antimicrobial materials studies, larger adverse effects such as the release of antimicrobial moieties via polymer biodegradation and the deactivation of antimicrobial effect over time due to the build-up of biological material may be masked [9]. The shortcomings of these contemporary methods are further exacerbated by the lack of validation extending beyond *in vitro* methods, as there have been minimal attempts to study new materials *in vivo* in their direct oral application [7]. In place of animal modeling, validation work has frequently moved directly into short *in situ* human studies (often labelling them clinical trials) to evaluate new materials’ anti-biofilm effect over the course of a few hours or days using specimens in a removable appliance [10,11]. There is currently a need for more physiologically-emulative methods that more strongly make the case of restorative efficacy and safety and pave the way for clinical study and adoption.

There is a long history of assessing dental materials and techniques on rodents [12,13], including as a model for caries formation when inoculated orally with a cariogenic bacterial species [14–21]. However, these caries and restoration studies are generally used to analyze the systemic or non-localized effects of treatments (such as a mouth rinse on the development of caries), or are used to first induce caries to apply a non-restorative or unbonded material treatment without removing caries [19,21]. The highly localized nature of antimicrobial restorative materials allows for a split mouth study where different restorations are placed on different teeth in the same animal to study treatment effects in the same environment [22–24]. One study published by Wu et al. in 2018 utilized a rat secondary caries model to evaluate an antimicrobial adhesive system [25]; however, this study had several important limitations. Cavities were restored using only an adhesive and not a total restorative system (dentin-adhesive-composite sandwich structure) used clinically, and caries analysis was conducted in 2D via tooth sectioning and scoring, or along 1-dimension at an evaluator-selected point to analyse mineral content via micro-computed tomography. This leaves a gap in dental materials testing methods between *in vitro* and human clinical where a more rigorous small-animal model may be suitable to expose new materials to a more challenging environment of enzymatic, bacterial, immunological and mechanical stressors simultaneously while collecting biocompatibility insights.

Previously, our research program has developed several techniques that we believe begin to accurately capture the complexity of the oral environment, combining exposure of new materials to simulated human salivary enzyme (SHSE) media that mimic the degradation of polymeric resins in the mouth and at the restoration margin, and detailed analysis of the long-term chemical and mechanical behavior of these materials [26,27]. We have also utilized confocal laser scanning microscopy to investigate the restoration margins and analyze bacterial penetration *in vitro* [28].

Our research group has also previously developed long-term antibacterial and anti-biodegradative materials based on novel filler particles for dental materials. These filler particles are synthesized through a mesoporous-silica-like process, whereby silica condenses around the water-facing surface of tubular antimicrobial (octenidine dihydrochloride, OCT) micelles and subsequently grows throughout the reaction into spherical porous silica particles with pores completely impregnated with antimicrobial. The extremely close non-covalent interaction between the resultant silica and antimicrobial drug molecules, as well as the approximately 35 % wt. loading of antimicrobial, results in a much more stable and long antimicrobial release period, without the destruction of the silica particle and the formation of voids [29]. These co-assembled drug-silica particles (DSPs) may then be added as a filler particle to resin dental materials to provide a reservoir of antimicrobial compound, stabilizing it within the resin matrix, and releasing it slowly or in response to bacterial enzymatic degradation without compromising the dental material’s basic properties [26]. Our past work developing a total-etch adhesive component incorporating 10 % of this filler system exhibited unprecedented long-term antimicrobial release, anti-biofilm capability, and an anti-biodegradative bond-preserving effect resulting from OCT’s inhibitory effect on salivary enzymes[27]. This novel bioactive adhesive requires more thorough analysis of its bioactive potential, providing the impetus for the current study.

Herein we aim to progress our evaluation of this novel filled-adhesive system to *in vitro* and *in vivo* (rat) anti-secondary-caries studies, as well as conduct a detailed *in vitro* analysis of bacterial penetration into the bonded interface. We hypothesize that the antimicrobial and anti-degradative effects of the material seen previously will translate to the current more elaborate evaluations, and that the novel antimicrobial-adhesive will result in significant decreases in bacterial biomarkers and markers of caries formation *in vitro* and *in vivo*.

## 2. Methods

### 2.1. Total etch adhesive material preparation

DSPs including the antimicrobial OCT at approximately 35 % by weight, were prepared as described previously [26], and mixed at 10 % final weight into the adhesive component of a total-etch adhesive system (Adper Scotchbond MP, 3 M, London, Canada) at 350 RPM for 24 h in total darkness (DSP-SBMP) [26]. This test material was used in all subsequent studies below.

### 2.2. In vitro bacterial biofilm model

#### 2.2.1. Specimen preparation

Intact extracted human 3rd molars were used (University of Toronto Human Ethics Protocol #32320), and dentin structure was prepared with a water-cooled low-speed diamond saw (Buehler Ltd., Lake Bluff, IL) exposing a minimum 3 × 3 mm surface at the cervical plane. Specimens were then bonded to commercial resin composite (Z250, 3 M, London, Canada) with “DSP-SBMP” or DSP-free “SBMP” total etch adhesive system (modified adhesive and as-provided etchant and primer components) according to manufacturer instructions (3-step process). Briefly: etchant gel was applied to the dentin slab top surface for 15 s and rinsed with DI water followed by blotting with laboratory wipes to remove excess water, exposing dentin collagen fibrils; a microbrush was used to apply primer and dried for 5 s with a gentle airflow; test or control adhesive was applied by microbrush and photopolymerized for 10 s at a distance of approximately 1 mm and a minimum intensity of 1730 mW cm^−2^ as verified by the device’s internal radiometer (Sapphire Plus Plasma Arc Curing System, Dent Mat, Santa Maria, CA); 2 mm of resin composite was applied and photopolymerized for 20 s as above; the dentin-adhesive-composite “sandwich” was then harvested by making vertical cuts with the low-speed saw producing a 3 × 3 mm block, which was then freed from the tooth structure with a horizontal cut leaving 3–4 mm of dentin attached to the specimen. Only the resin-dentin interface was exposed to bacteria and exogenous enzymes by sealing the adjacent dentin with clear nail varnish [28,30]. The prepared specimens were sterilized with gamma irradiation at 25KGy known not to alter the interface properties [28,30].

#### 2.2.2. In vitro biodegradation and chemostat biofilm model

Specimens were preincubated in SHSE media according to protocol [26], mimicking physiological oral esterase-like biodegradative activity, (37°C, pH=7) for 0-, 180-days to artificially age and induce the specimens’ interfacial biodegradation (N = 3/group/time). Previous studies of *in vitro* biomarkers at the restoration-dentin margin indicate adequate power to detect moderate effect sizes (α error 0.05, 1-β error 0.8) [28,31].

After aging, specimens were aseptically transferred and suspended in a chemostat-based-biofilm-fermenter (CBBF) containing fresh medium (1/4 × Todd Hewitt-Yeast extract media supplemented with 10 mM sucrose) and inoculated with *Streptococcus mutans* UA159 and *Lactobacillus rhamnosus* ATCC 11981, generating a synergistic and acid-producing biofilm that would mimic intraoral pathogenic growth conditions for 3-days [28,32]. Incubation media pH was maintained at pH 7.0, using a 0.5 M KOH or HCl buffering solution, and temperature was maintained at 37°C. Fresh medium was pumped into the vessel at a rate of 5.7 L/day (dilution rate of D=0.6/hour), mimicking human resting salivary flow rate [33,34].

#### 2.2.3. Confocal microscopy analysis of in vitro marginal biofilms

Following incubation, specimens were removed and rinsed with PBS and stained by BacLight Live/Dead Viability Kit (Molecular Probes, Eugene, Oregon, USA). Z-stack images of the resin-dentin interface were captured along 3 sides of each specimen through a 63x/1.4NA oil objective lens, using confocal-laser-scanning-microscopy (CLSM) (Leica SP8 Confocal Microscope, Wetzlar, Germany). CLSM analysis started immediately at the subsurface of the sample to a depth where no bacteria were detectable, capturing images at 0.5 μm intervals as described previously [28]. The marginal interface and gap (if any) was defined as the interface between the resin restorative material and dentinal regions, and all regions of interest were standardized for orientation. Z-stacks were then analyzed by quantitative software (Imaris version 9.1, Bitplane AG, Zurich, Switzerland) for biofilm biomass (live and dead cell counts), viability, and z-depth of interfacial bacterial penetration. Background fluorescence of unstained and stained non-incubated specimens was used to control for autofluorescence.

### 2.3. In vitro secondary caries protocol

#### 2.3.1. Secondary caries static incubation protocol

Experimental specimens were prepared as in Section 2.2.1 (N = 3/group) and were incubated statically for a total of 7-days in four 225 mL flasks containing Trypticase Soy Broth-Yeast Extract media (TSB-YE), supplemented with 2 % sucrose and 1 % glucose, and inoculated with cultures of *S. mutans* UA159 and *L. rhamnosus* ATCC 11981 grown from frozen stock in TSB-YE for 18 h (5 % CO_2_ at 37°C)[35]. Specimens were transferred to a flask with new media and inoculated with fresh primary cultures every 48 h of incubation (5 % CO_2_ at 37°C). Following incubation, specimens were removed and rinsed with PBS.

#### 2.3.2. Micro-computed tomography analysis of in vitro secondary caries

Volume of demineralization and cavitation were measured using micro-computed tomography (μCT) (Phoenix v|tome|x microfocus CT, GE, Wunstorf, Germany) (voltage at 80 kV, 5.92 μm voxel size) and quantitative software (Medical Imaging Toolkit version 2018.04.2, German Cancer Research Center, Heidelberg, Germany). The resin-dentin interface of each specimen was identified on sagittal and coronal views, and the corresponding axial view was used to quantify demineralization and cavitation areas. The area adjacent to the resin-dentin interface was evaluated (21.25 μm above and 85 μm below the interface, towards the resin and dentin respectively; 106.25 μm in total z-distance). The expected area of intact dentin was measured as the area of resin 21.25 μm above the interface, representing the original specimen cross-sectional area. Cross-sectional images of remaining dentin were then produced at 21.25 μm, 42.5 μm, 63.75 μm and 85 μm from the interface on the x-y plane. Cavitation volume (volume of dentin structure lost) was calculated as the resin cross-sectional area minus an individual dentin cross-sectional area, multiplied by the distance between cross sections (21.25 μm), and then summed across the 4 cross-sections to produce an approximate volume. Demineralized area (area with visually identifiable loss of mineral density) was calculated by measuring the area of each cross-section scan with an intensity of between 35 % and 45 % that of the intensity at the centre of the dentin (in practice the demineralized perimeter of the dentin slab was readily identifiable in cross-sectional scans). Demineralized volume was then taken as the sum of demineralized areas (4 per specimen) multiplied by 85 μm.

### 2.4. In vivo secondary caries model

#### 2.4.1. Animal surgeries and care

8 male and 8 female Sprague-Dawley rats purchased from Charles River (SAS Sprague Dawley Rat) with weights 250–350 g (approximately 13 weeks in age) were used for the study (University of Toronto Animal Ethics Protocol #20012382) [12,13,36]. Animal groups were randomly assigned based on equal numbers of each sex in each group and consisted of: Split-mouth Test (T, 2 antimicrobial DSP-SBMP test restorations and 2 control SBMP adhesive restorations each, assigned randomly left or right), and Vehicle Control (VC, 4 control SBMP adhesive restorations each), allowing a moderate DSP-SBMP effect size across studies of d_z_ = 0.65 (α error 0.05, 1-β error 0.8).

Animals were anaesthetized with isoflurane via nose cone, the jaw retracted, and 0.15 mL 3 % carbocaine with 1:100,000 Epinephrine was administered to the mucosa. A 0.5 mm diameter carbide bur was used to create two 0.3 mm deep and 0.5 mm wide semi-spherical defects, one each in the mesial and lingual enamel and dentin of the left and right 1st maxillary molars (4 total). The defects were restored according to manufacturer instructions using either the DSP-SBMP or SBMP total-etch adhesive systems described above in a material-blinded manner (etch, rinse, primer, and adhesive steps), and subsequent resin composite application to match the original tooth geometry (Filtek Supreme Ultra Flowable, 3 M, London, ON). Animals were inoculated with 0.1 mL of log-phase *S. mutans* UA159 and *L. rhamnosus* ATCC 11981 in Todd Hewitt + 0.3 % yeast extract broth via oral swab to promote cariogenic bacterial growth [32].

10 % sucrose water and mashed feed was provided *ad libitum*. For the first day post-operatively, bacterial inoculum broth (10 mL) was added to 90 mL of sucrose drinking water and provided *ad libitum*. Dentition was visually inspected at 3-weeks under isoflurane anaesthesia and sacrifice occurred 7-weeks post-op via CO_2_ asphyxiation and cervical dislocation. Necropsy was performed and animal maxilla with teeth intact, liver, kidneys and GI tract from stomach to large intestine were fixed in 10 % neutral buffered formalin for 24 h, washed in 70 % ethanol for 24 h, and stored in PBS.

#### 2.4.2. In vivo clinical caries observation and scoring

At the 3- and 7-week examination points, maxillary dentition was examined (by optical microscope after necropsy) and caries on the 1st and 2nd molars was scored visually by a blinded dentist without knowledge of the animal group using the International Caries Classification and Management System (ICCMS^™^)[37]. Restoration retention was also noted.

#### 2.4.3. Pathology and biocompatibility analysis

After necropsy, liver, small and large intestines, and kidneys sections from T and VC groups were grossed, mounted in paraffin, sectioned (5 μm), and stained by hematoxylin and eosin stain by a trained histologist. These sections were examined blindly by a pathologist (author D. A.) with a routine light microscope (N = 3/site/animal). Observations were scored as absent (0) or present (1), or; absent (0), minimal (1) mild (2) and moderate (3), where appropriate. Statistically different results were reported on.

### 2.5. Statistical analysis

Statistical analysis was performed using SPSS 24 (IBM Inc., Chicago, IL, USA); in all tests, significance was defined as p < 0.05 and homogeneity of variance and normality of data was confirmed when appropriate using Levene’s and Shapiro-Wilk’s tests, respectively, prior to further analysis. Mean data of normal distributions are presented with ± one standard deviation. Two-way analysis of variance (ANOVA) and Tukey’s HSD multiple comparison tests were performed to determine the main effects of DSPs and time on interfacial biomass, viability, and penetration. Unequal variances T-test was performed to evaluate the effect of DSPs on *in vitro* interfacial demineralization and cavitation. *In vivo* clinical animal observations and restoration retention rates were analysed via paired T-test and across T animals via contingency table (χ^2^) analysis with Fisher’s exactness where appropriate.

## 3. Results

### 3.1. In vitro marginal biofilm analysis

**Fig. 1.**
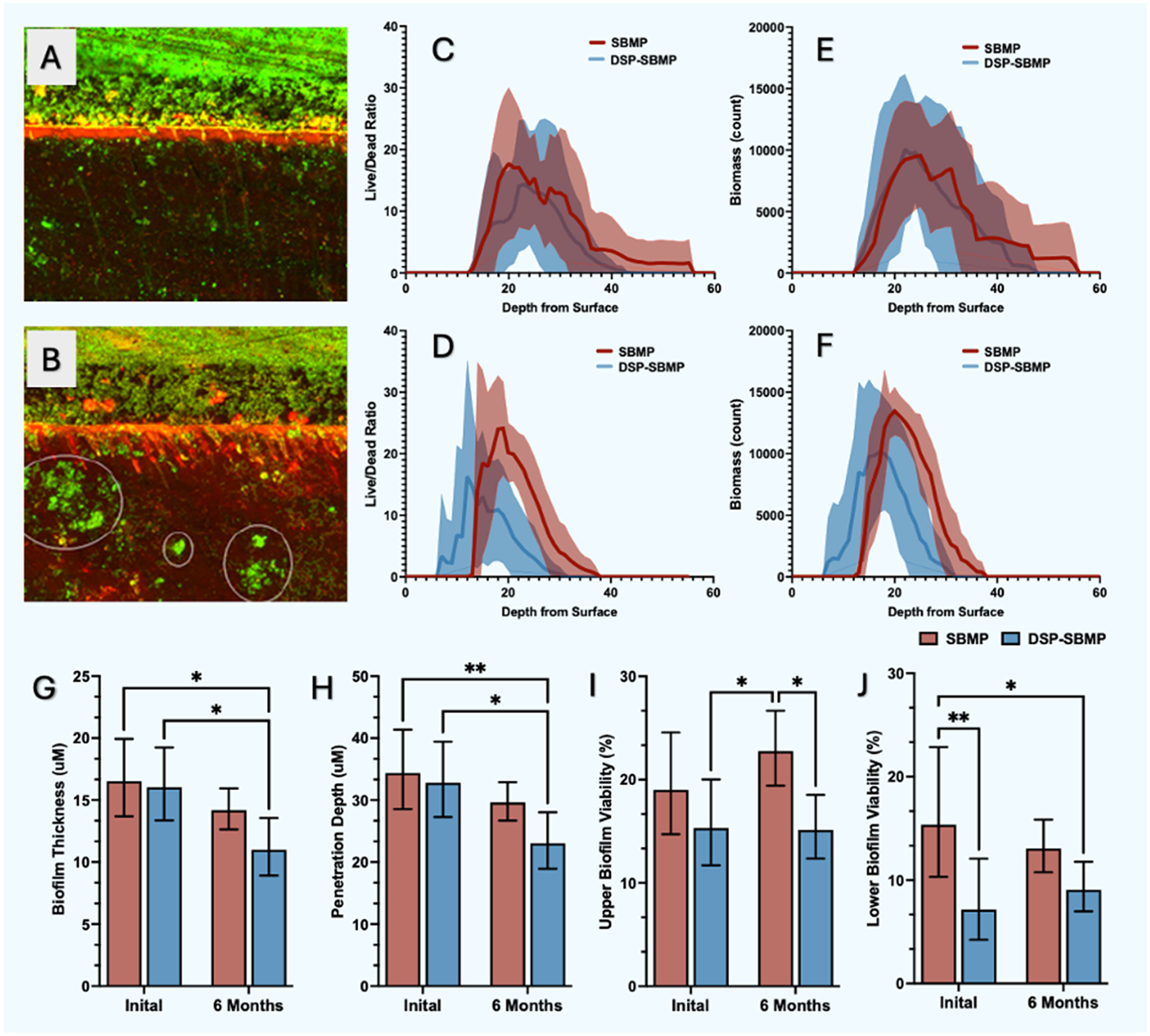
(A-B) Representative confocal laser scanning microscopy images of *S. mutans* and *L. rhamnosus* biofilm formed within the interface of (A) DSP-SBMP and (B) SBMP after 180 days in SHSE (live cells in green, dead cells in red/orange). Representative large clusters of live cells on SBMP samples are circled in (B). (C-D) Bacterial viability and (E-F) bacterial biomass (cell count) at each z-depth from the SBMP control or DSP-SBMP test interface surfaces after 0 days (C,E) and 180 days (D,F) pre-aging in SHSE where bacterial biomass and the ratio live and dead cells is indicated by the blue and red areas with lighter shaded regions demonstrating ± standard deviation, for DSP-SBMP and SBMP, respectively (N = 9 per depth value per group) with. depth of penetration is shown along the x-axis. Extrapolated bacterial quantification included (G) total biofilm thickness, (H) penetration depth, as well as viability at the upper (I) and (J) lower 10 μM of interface depth. *p < 0.05, **p < 0.01.

Fig. 1A–B displays representative CLSM micrographs depicting the antimicrobial effect of DSPs on biomass formation and viability in SBMP adhesives, with thicker biofilm formation and more live cells on the surface of the interface of control SBMP vs. DSP-SBMP specimens. DSPSBMP specimens on average experienced significantly (p < 0.05) reduced total biofilm biomass (cell counts) (-19.2 ± 4.9 %) and viability (-23.1 ± 4.3 %) throughout the artificially compromised adhesive interface (Fig. 1 C–F). Additionally, only the DSP-SBMP group resulted in a significant (p < 0.05) reduction in biofilm thickness (27.1 ± 8.2 %), total penetration depth (-15.5 ± 5.6 %), and viability in deep biofilm sections (-50.4 ± 13.5 %) biofilm following 180 days pre-incubation (Fig. 1 G–J).

### 3.2. In vitro secondary caries formation

**Fig. 2.**
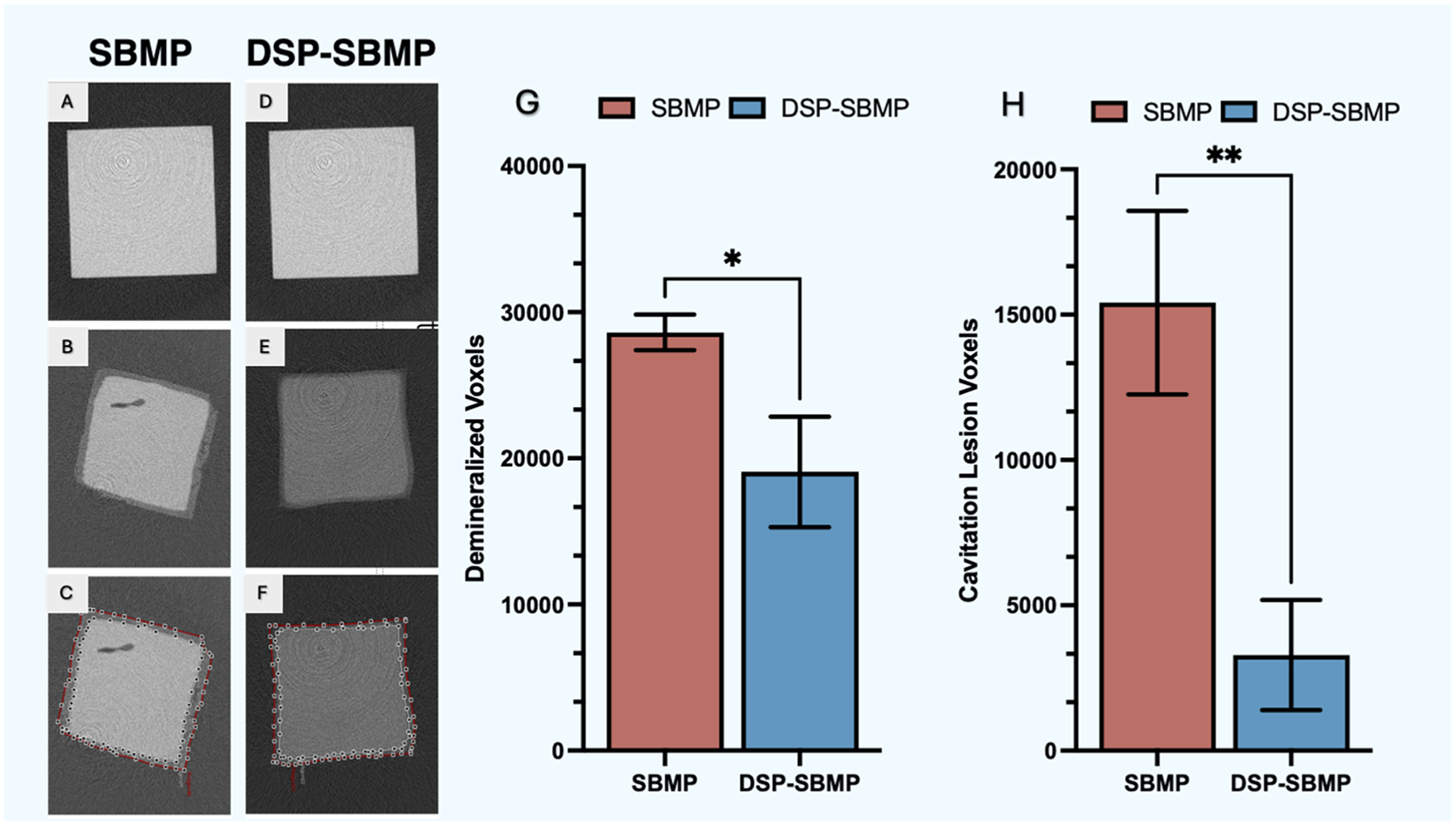
(A-F) Axial (top-down) view of μCT scans of representative (A-C) SBMP and (D-F) DSP-SBMP composite-adhesive-dentin specimens following 7 days incubation in dual species biofilm, where image plane is parallel to the bonded interface. (A,D) Intact resin, 21.25 μm above the restoration-dentin interface; (B,E) demineralized/cavitated dentin, 42.5 μm below the interface; and (C,F) area of demineralized dentin is calculated by subtracting areas of the white outlined shape (highly mineralized dentin) and the red outlined shape (total dentin). Cavitated area was calculated by subtracting the area of total dentin (red outlined shape) from the original sample area represented by the intact resin area in (A). Average (G) demineralized and (H) cavitated voxel volumes of SBMP and DSP-SBMP specimens. N = 9, *p < 0.05, **p < 0.01.

Representative μCT images comparing SBMP and DSP-SBMP specimens following exposure to dual species biofilm are displayed in in Fig. 2 A–F. All non-incubated control specimens showed no differences in demineralization and cavitation volumes (p > 0.05). Following 7-days incubation with *S. mutans/L. rhamnosus* co-culture, the volume of demineralization was 0.085 ± 0.004 mm^3^ for control unmodified SBMP specimens and 0.057 ± 0.011 mm^3^ for DSP-SBMP specimens, which is calculated to an average reduction in demineralization of 33.4 ± 8 % for DSP-SBMP (p < 0.05, Fig. 2 G). Cavitation volumes for control unmodified SBMP and DSP-SBMP specimens were 0.046 ± 0.009 mm^3^ and 0.010 ± 0.006 mm^3^, respectively (p < 0.05, Fig. 2 H). The average reduction in cavitation was 78.6 ± 13.8 % for DSP-SBMP specimens.

### 3.3. In vivo clinical caries observations

**Fig. 3.**
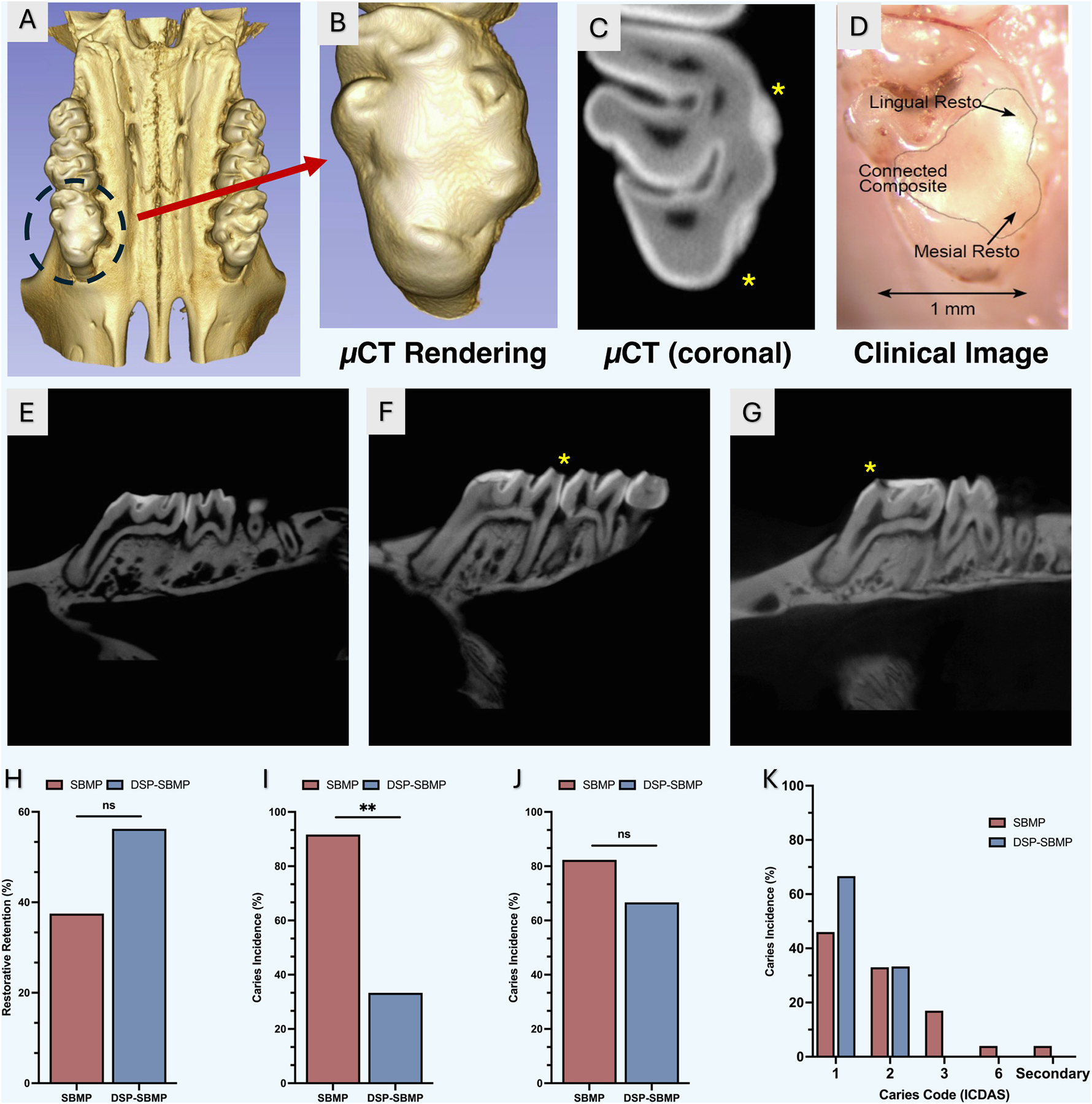
(A-B) Sample μCT renderings, (C) adhesive mesial and lingual bonding sites and (D) clinical diagram of representative connected composite restoration. (E-G) Representative μCT visualization of restored molars presenting (E) healthy status, (F) shallow caries, or (G) secondary caries. (H) Total restorative retention for SBMP and DSP-SBMP groups. (I) Average caries incidence on restored molars and (J) all teeth (whole mouth effect). (K) Caries incidence grouped clinical observation of carious lesion type based on the International Caries Detection and Assessment (ICDAS) score. N = 16, *p < 0.05, **p < 0.01.

Animal surgery successfully created and restored sub-millimeter-size standardised defects using a 3-step total etch adhesive and flowable composite despite the confined working space and size of cavitation/restoration. Clinically during restoration preparation, the flowable composite was allowed to spread from the mesial to lingual surface during application and allowed to connect. The orientation and the low viscosity/flowability of the composite made keeping the applied restorations separate when in close proximity, and the increased amount of composite used and increased contact surface area was believed to potentially distribute masticatory forces across the tooth as well as bonded interface, potentially increasing retention (Fig. 3 A, B, D). However both defects’ surfaces were prepared with adhesive at the same time meaning restorations were separately bonded on both defect sites, but not bonded to the areas between the defect sites covered by composite (highlighted in Fig. 3 D). A μCT slice demonstrating a typical restored 1st molar is presented in Fig. 3 C with the two bonded surfaces visible. This increased to some extent the variability of total restoration size and bonded surface area. Despite these challenges surgeries were performed successfully, except for one DSP-SBMP group animal (A11) that had pre-existing caries on maxillary 2nd molars, and exhibited nociceptive behavior post-operatively, managed with additional meloxicam to reduce pain.

Most animals were observed with some initial caries (ICDAS Code 1 and 2) at the 3-week interim check-up, with rampant caries observed in all animals at the 7-week endpoint. Although DSP-SBMP restorations had more intact restorations (56 %) compared to SBMP restorations (37 %) in split mouth animals, the results were not significant (Fig. 3 H (χ^2^ p > 0.05 and split mouth paired *t*-test p > 0.05). The low retention among both groups could be due to several external biological or mechanical factors, especially considering the animals’ constant chewing behavior on objects in their environment, and thus data from teeth with lost restorations were excluded from analysis. Across all split-mouth animals there was a statistically significant reduction in the total count and ICDAS scores of caries on whole-teeth restored using DSPSBMP adhesive by clinical post-mortem observation, and a significantly higher rate of caries-free diagnosis (χ^2^ p < 0.05, Fig. 3 I, K,). Interestingly, this effect did not translate to a whole mouth reduction in caries as determined by comparing total scores between T and C animals (χ^2^ p > 0.05, Fig. 3 J), suggesting a potential significant but highly localized reduction in bacterial reservoir that may affect only the relevant restored tooth. Secondary caries was only counted (and indeed were only observed) at the bonded interface of the restoration and not under the non-bonded flowable composite between the bonded sites, avoiding any confounding effect this excess-applied area of composite may have had.

### 3.4. In vivo biocompatibility

Histological sections of animal kidneys, liver and large and small intestine were analysed while blinded to group and compared between SBMP and DSP-SBMP animal groups and in the context of potential pathology or toxicity [38,39]. Histological evaluation of small and large intestine sections were unremarkable, displaying no differences between experimental groups (p > 0.05, data not shown). Histological results for liver and kidney sections are described below.

#### Liver:

Analyses of liver tissue of control and treatment animals were based on the comparison of the following parameters: the presence of portal and lobular cellular infiltrate (absent, present; minimal, and mild), macrovesicular and microvesicular fatty change (absent <5 %, mild 5 %-33 %, moderate 33 %-66 %), biliary injury (absent/present), bile pigment deposition i.e. cholestasis (absent/present), necrosis and/or apoptosis (absent/present), clear cell change (absent, present; mild, moderate), extramedullary hematopoiesis (absent/present), and other findings as intracytoplasmic inclusions/other pigments.

**Fig. 4.**
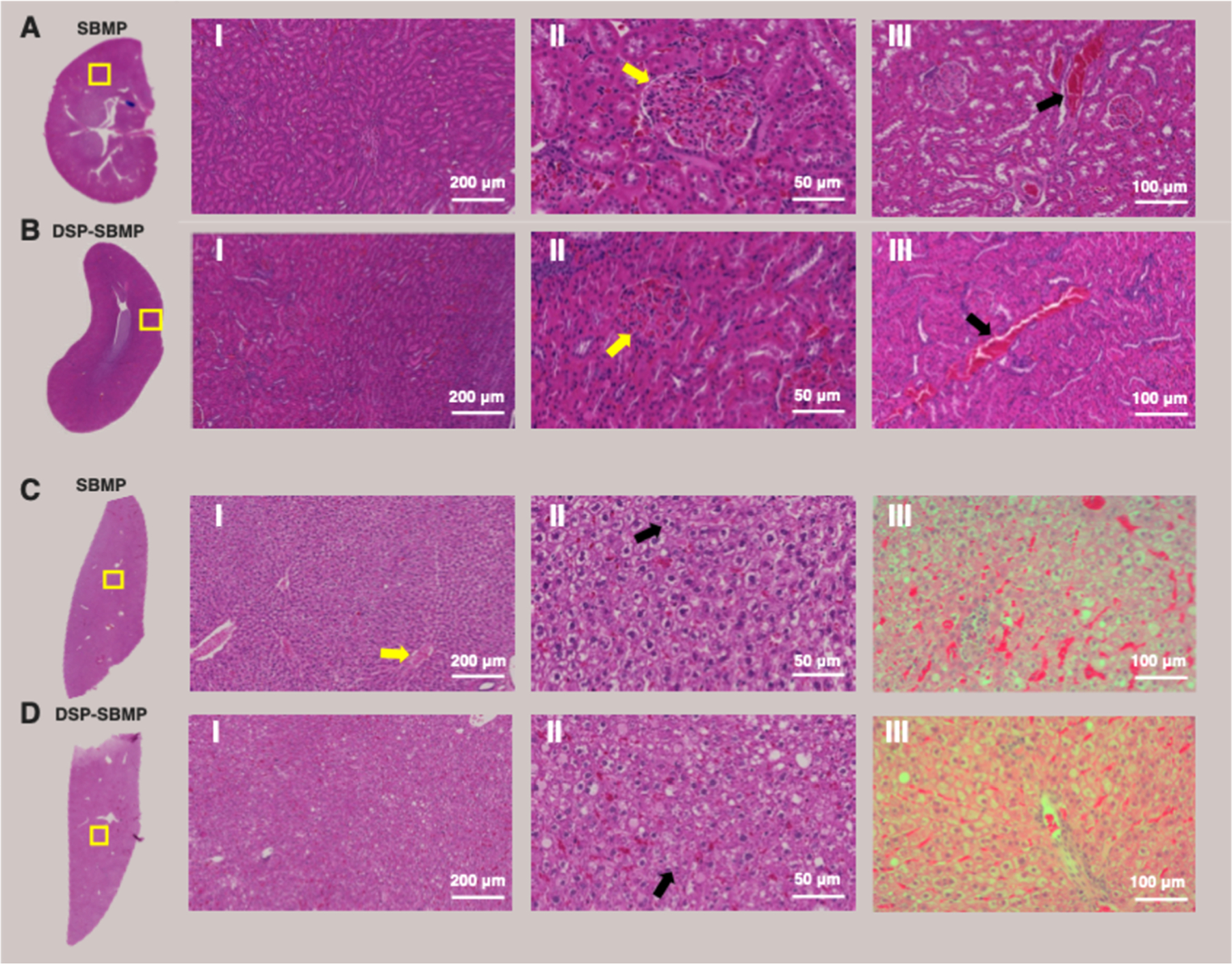
Histological micrographs of representative kidney **(A, B)** and liver **(C, D)** cross-sections for control (SBMP) and experimental (DSP-SBMP) adhesive animals (H&E). **(A, B)** Yellow arrows illustrate glomeruli and Bowman’s capsule and black arrows representing blood sinusoids of collection ducts in kidney sections. **(C, D)** The yellow arrow illustrates an instance of periportal microvesicular steatosis, and the black arrows illustrate hepatocytes in liver sections. Liver image set III has not been stained differently, but has been digitally processed (whole-image hue, saturation)to allow for the differentiation of the presence of lobular cellular infiltrates with macrophages and pigment.

Minimal to mild lobular and portal cellular infiltrates were observed in 10/16 (4 DSP-SBMP and 5 SBMP) liver histology sections. Most were characterized as lymphocytic infiltrates except in two cases (both DSPSBMP), which showed mixed infiltrates (lymphocytes, neutrophils, and eosinophils). None of these infiltrates found (which were all focal) were associated with significant parenchymal injury or necrosis. Tiny foci of extramedullary hematopoiesis were identified in 4/16 animals (2 DSP-SBMP and 2 SBMP). Macrovesicular steatosis (mild) was observed in 3/16 animals (2 DSP-SBMP and 1 SBMP), distributed in a zonal pattern in all cases. Mild microvesicular steatosis (Fig. 4 CI) was observed in one animal (SMBP)with periportal and mid-lobular zonal distribution. Pigment (bile deposition; cholestasis) and minimal cholestasis was seen in 4/16 animals (2 DSP-SBMP and 2 SBMP), however, none of these cases were associated with bile duct injury, bile duct reaction, or ductopenia. Clear cell change (glycogen accumulation) was seen in all the rodent’s liver sections, ranging from mild to moderate cytoplasmic clearing. Other findings included hyaline material inclusion in some of the hepatocytes, mostly representing plasma cell influx which is an artifact.

#### Kidney:

Analyses of renal tissue of control and treatment animals were based on the comparison of the following parameters: glomerular changes (0 =absent, 1 =present), tubulointerestitial cellular infiltrates (0 =absent, minimal=1, mild =2, moderate=3), hilum changes/urothelial inflammation (absent=0, mild=1, dense=2), the presence of mineralized material (absent=0, present=1), and vascular changes (0 =absent, 1 =present).

There were no histological changes in the presence of glomeruli (Fig. 4ABII) or blood sinusoids (Fig. 4 ABIII) between groups. Cellular infiltrates were also found across animals with no statistical differences between DSP-SBMP and SBMP animals (p > 0.05). Mineralized/calcified material was found in 1/16 animals (DSP-SBMP) with a very focal and mild tubular epithelial injury. No associated necrosis was present, and no linear deposits of papillary mineralization were seen, suggesting that these deposits have no pathologic significance. Interstitial cellular infiltrates were classified as minimal to moderate for all test animals with no differences observed between groups (p > 0.05). These infiltrates are usually composed of lymphoid or mononuclear cells, but other inflammatory cell types may also be seen. Moderate findings are considered a routine in the kidney and can be age-related. In many cases, they represent a background finding that has little pathologic significance. Cellular infiltrates are different from inflammation in that the number of inflammatory cells is relatively low, and no other inflammation indicators (e.g., edema, hemorrhage, tissue damage) are present.

## 4. Discussion

This study represents a thorough pre-clinical analysis of the performance of a novel antimicrobial dental adhesive system for the inhibition of marginal degradation and secondary caries. The analysis and results described here, combined with previous studies of the novel restorative’s properties [26,27,29], allow for a mechanistic understanding of DSPs’ antimicrobial properties and their role in the maintenance and integrity of the bonded interface.

### 4.1. The effect of DSPs on bacterial biodegradation markers

The inclusion of DSPs in SBMP total-etch adhesive component effectively reduced bacterial biomass and viability over 6 months, demonstrating a strong, prolonged capability of local antimicrobial therapy to reduce the levels of marginal bacteria and maintenance of inhibitory concentrations of the antimicrobial/antidegradative agent OCT long-term. These results agree with the initial investigations of the mechanism of DSP and antimicrobial OCT release with modeled long-term efficacy at the restoration-tooth interface [26]. The current study further challenged DSP-adhesive efficacy by subjecting the material *in situ* to 180-days preincubation in biodegradative SHSE media, triggering initial burst- and a large amount of steady-state-release of antimicrobial during adhesive biodegradation, from a significantly lower mass of adhesive placed (as intended) as a thin adhesive layer instead of a large bulk specimen used in the previous study. Subsequent exaggerated 3-day cariogenic bacterial biofilm challenge confirmed antimicrobial efficacy and results show consistent biofilm reduction at 0-and 180-days, implying that OCT release remained at sufficiently high levels to disrupt biofilms. However, the biomarker protocol and CLSM analysis was not able to discern whether bacteria were prevented from colonizing the exposed adhesive surface, inhibited (but not completely eliminated) away from the surface, or most likely, some combination of these two effects as released OCT leeches from the surface through the margins with other restorative material by-products. These results complement previous modeling based on 90-day data showing a highly localized steady-state antimicrobial release from the adhesive surface that does not change greatly in magnitude over time. In the previously developed model of antimicrobial release [26], OCT diffusion from the adhesive follows a square-root-of-time profile until its release half-life. Considering the adhesive layer in this previous report [26] and the present study are of similar geometries with low exposed surface areas at the restoration perimeter and very low rates of OCT release, total release as a portion of payload would not exceed 5 % after 10 years, and rate of release will not have significantly declined in this time. This indicates similar antimicrobial efficacy at 10 years as observed at 180 days, and provides a strong rationale for long-term anti-caries potential through the 5–7 period most strongly associated with secondary caries formation [40,41].

The results of the current study agree with those of Huang and Kermanshahi [28,30], who demonstrated increasing maximum bacterial mono-species biofilm penetration with incubation time. The current study utilized a dual-species model for biofilm formation with *S. mutans* and *L. rhamnosus*, rather than *S. mutans* alone, and incubated over 3 days instead of 7 as before. *L. rhamnosus* has a similar cariogenic potential to *S. mutans*, but together these species have synergistic cariogenic effects [32,42], which may increase the destruction of the restoration margin by acid production, increasing the ability of individual cells to penetrate deep in the margin with or without prior polymer matrix biodegradation by SHSE. The maximum depth of penetration seen in 180-day incubated specimens in the current study is similar to those seen after 90–180 days of SHSE preincubation in previous mono-species studies [28,30].

Depth of bacterial penetration may be a good indicator of marginal breakdown, however, as hypothesized previously by Huang et al., bacterial adhesion and biofilm formation may be more critical in predicting secondary caries development, and therefore restoration longevity [28]. Marginal breakdown in the first experiment, measured as depth of bacterial ingress in the current study, does not necessarily represent the formation of secondary caries, since the majority of interfacial degradation is by enzymes in SHSE through an incubation period of up to 180 days, rather than the 3-day exposure to bacterial acid and enzymes in the current experiment. This biodegradative breakdown of the resin is more likely a modulating factor in cariogenic biofilm activity.

### 4.2. The effect of DSPs on in vitro secondary caries development

The incorporation of antimicrobial DSPs into the dental adhesive systems was highly effective in inhibiting interfacial dentin mineral loss, cavitation, and therefore reducing artificial secondary caries in this short-term *in vitro* model. This model did not simply mimic the physiological conditions of the oral cavity but instead provided a greater pathogenic and cariogenic challenge leading to observable damage over a short timeframe, due to uncontrolled pH and a renewing nutrient supply. Demineralization and cavitation, whether quantified as volumes or observed clinically via radiography, accurately represent secondary caries development and are clinically relevant outcomes that relate directly to clinical decision making by dental practitioners in diagnosing secondary caries determining whether a restoration needs to be replaced or not.

The reduction in cavitation volume was approximately twice as much as demineralization, agreeing with clinical observations of a 2-step process, where tissue is first demineralized and subsequently exposed organic material is removed, and a cavity forms. The presence of DSPs appears to significantly slow both processes, thus preventing the progress of tissue from the “demineralized” phase to the “cavitated” phase of caries progression. There are two purported mechanisms in which DSPs and OCT work to preserve the restoration interface and maintain marginal integrity. Firstly, OCT’s broad antimicrobial efficacy against gram positive and negative bacteria prevents secondary caries by reducing cariogenic bacteria biofilms, as supported directly in this study, and therefore cellular metabolism and production of acid by gross reduction of biomass. Secondly, OCT released from DSPs inhibits enzymatic biodegradation of the adhesive resin matrix by human salivary and bacterial enzymes [27], slowing the release of biodegradation by-products such as bisHPPP that upregulate virulence factors in oral pathogens [43,44]. It could be hypothesized that if the antimicrobial and anti-degradative activity of dental adhesives containing DSPs are effective in the extreme conditions of the current study, they would likely be effective in the oral cavity, where the buffering capacity of saliva, fluoride exposure, salivary dilution, and mechanical plaque removal are present to slow the development of caries over a longer period [45].

### 4.3. Effect of DSPs on secondary caries in vivo

Although there is a strong history of both caries investigations [14–18] and restoration material efficacy and toxicity modeling using rodent models [12,13], there is only one predicate known to the authors for the study of secondary caries in rodents [25]. The present study was successful at inducing primary and secondary caries in all animals, including caries readily observable by clinical analysis. Furthermore, despite the challenges of the animal surgery, the procedures were completed using a full clinical-style process of cavity (defect) preparation, etching, priming, bonding and build up with a flowable composite. Tooth restoration was challenging in this environment, and the use of a flowable composite, intended to ease application through use of a blunt needle, may have complicated restoration by flowing away from the intended site before curing. While he unbonded contact between flowable composite and intact enamel between defect sites did not lead to observed secondary caries in this study, it is unclear what effect this had, if any, on restoration retention. Future studies should consider a less-flowable bulk fill composite material during restoration. The presence of only a thin layer of adhesive material covered by a non-bioactive composite is identical to the clinical situation, and anti-caries performance should more closely match the clinical situation than the predicate study’s use of adhesive-only to build a restoration.

The use of DSP-SBMP in animal restorations was directly associated with less severe, or a lack of, caries on the same tooth (maxillary first molar) across split-mouth T group animals, as observed by post-mortem clinical observation and ICCMS/ICDAS caries scoring criteria. Considering the challenging environment for the restoration and tooth, with cariogenic bacteria seeded on its surface and near constant sucrose supply, this is a very positive result. Since *in vitro* data clearly showed that the presence of DSPs reduced cariogenic biofilm formation, it can be assumed that this effect persisted *in vivo*. The release of OCT from DSPSBMP would have been significantly lower than levels needed to achieve a salivary inhibitory concentration in the rat mouth due to the limited exposed adhesive at the restoration margin and constant salivary dilution [26], and is supported by the lack of whole-mouth effect (T versus C animal group effect). Therefore, derived protection of the adjacent tooth may be due to a decrease in the reservoir of cariogenic bacteria through a reduction in marginal biofilm. Rat molars are in constant use and it is well understood that cariogenic biofilms typically do not form and induce caries on their molars’ smooth surfaces [46]. The margins around restorations may help accelerate caries formation in the present study by providing a sheltered area able to retain a cariogenic biofilm over the life of the animal. The presence of even low levels of OCT from the restoration may decrease the rate with which this reservoir biofilm is able to disperse to other nearby surfaces. This potential effect will require further analysis to confirm.

In general, the length of the study may have resulted in rampant primary and secondary caries that may have reduced the magnitude of observable effects of DSP-SBMP. Although secondary caries rat models have been previously conducted for 5 and 7 weeks [15,16], the previous secondary caries study by Wu et al. included only a 3-week incubation period [25]. Indeed, in the present study, caries was observed clinically in several animals at a 3-week midpoint exam. Caries generally should be induced in rats through some means, otherwise variables such as normal diet, housing, natural exposure to pathogens, age, weight, and strain of rat make underlying caries rates highly variable, limiting the power of the study [47]. At 7 weeks caries is easily diagnosed by simple optical microscope and probing instrument. But the high stress of rat masticatory activity leading to restoration loss, and the extremely challenging accelerated caries environment produced by constant sugar supply, ensures that this restoration margin “weak link” region is bombarded with cariogenic factors that overwhelm its ability to inhibit bacterial growth. In a more realistic human oral environment that isn’t under this high level of challenge, the antimicrobial benefit of DSP-adhesive will still likely increase the time needed for cariogenic factors to compound into secondary caries. Future *in vivo* analysis should focus on tuning the cariogenic challenge presented to the animals to optimize the usefulness of results.

### 4.4. Biocompatibility of DSP-adhesive

In this work, we undertook comprehensive multiorgan histology to evaluate the biocompatibility of both the control and experimental restoratives. DSP-SBMP presence had no significant systemic impacts in the health of most animals. The observed defects and foci identified in this study were not associated with toxic effect in the context of expected normal rat histopathology [38,39]. This result was largely expected; octenidine dihydrochloride has undergone extensive testing and is widely regarded as a safe antimicrobial when administered orally [48,49]. Furthermore, at the exposure concentrations experienced by animals in the current study which would be DSPs release from the exposed adhesive interface, the silica phase of DSPs is not expected to elicit an adverse reaction based on previous work analysing the toxicity of mesoporous silica [50].

The statistically significant increase in calcified material in renal histology slides from treated animals is interesting, but it was not clear from microscopic analysis that these foci were directly caused by DSPs, and the size of the foci was not consistent with the diameter of individual DSPs. Typically, mesoporous silica persists, if at all, in the liver [50], however persistence location may be highly dependant on particle size. Presence of renal mineralized foci was not associated with restoration loss versus retention. Furthermore, in only 1 case, the aforementioned T group animal A11, was the calcified material associated with a very focal and mild tubular epithelial injury. In no cases was it associated with necrosis or linear deposits of papillary mineralization. A11 also demonstrated cellular infiltrates that were significant, as they were more severe, multifocal, and associated with focal areas of mild edema, mineralization, and mild tubular epithelial changes (such as basophilia, nuclear apoptosis, and reactive mitosis).

### 4.5. Relationship of results to clinical outcomes

There is a positive relationship between the presence and abundance of cariogenic bacteria and caries formation by acid production [51]. There is also evidence that biodegradation, as a result of human-salivary, human-neutrophil, and bacterial enzymes, contribute to the development of secondary caries [3,4]. It can therefore be hypothesized that a reduction in biofilm formation, the viability of cariogenic bacteria, and the depth of bacterial ingress would correlate to a reduction in marginal breakdown and secondary caries development. Whether these reductions in bacterial biomarkers are clinically relevant and predictive of secondary caries formation under clinical settings are yet to be fully explored. However, the current study suggests that there is a close relationship between interfacial BBMs and formation of recurrent cavitation, as both were significantly reduced by the antimicrobial adhesives.

From these *in vitro* results we would expect the anti-caries capability to persist *in vivo*, although potentially with reduced efficacy owing to the broader microbiota diversity and increased number of factors potentially degrading the restoration-tooth interface (host immune response, temperature, mastication). The current study, despite these limiting factors, observed a clear anti-secondary caries effect of DSP-SBMP in rats, strongly indicating clinical relevancy. However, some of the observed effects, such as the “whole tooth” reduction in caries in DSP-SBMP restored teeth, may not be relevant to humans which have multiple locations on teeth to act as a cariogenic bacterial reservoir between cleanings.

The next step is to demonstrate broad biocompatibility with standardized 3rd party toxicity and sensitization studies recognized by regulatory bodies, and subsequently introduce the material in a clinical human restoration with a non-accelerated cariogenic challenge and monitor the effects over the period associated with secondary caries formation (5–7 years), or introduce to an at-risk population and monitor for a shorter period of time. The present data allow us to conclude that DSPs *likely* reduce bacterial biomarkers and marginal breakdown and demineralization clinically, but only clinical data can confirm the ability to prevent caries disease.

## 5. Conclusion

The current study successfully developed and utilized a comprehensive *in vitro* bacterial biomarker and artificial caries model, and an *in vivo* rat secondary caries model, to demonstrate the efficacy of a novel antimicrobial and anti-degradative dental adhesive in a clinically relevant application. The bacterial biomarker study supported previous *in vitro* results from more typical methods, and in turn were supported by *in vivo* data collected, meaning the methods may serve as a useful and more realistic *in vitro* method for analysing potential anti-caries materials. Previously observed antimicrobial effect was corelated with current reductions in bacterial viability and biomass. These minor reductions subsequently were associated with significantly larger reductions in demineralization and cavitation *in vitro*, effects that were observable *in vivo*. Work remains to expand the scope of analysis and tune the length of the *in vivo* study, but the work here represents a significant volume of evidence demonstrating efficacy of the developed material. Future work includes first use in humans and clinical study of the material’s performance and efficacy, both of which are critical to demonstrating the ability to prevent disease.
